# Facial Nerve Palsy: Providing Eye Comfort and Cosmesis

**DOI:** 10.4103/0974-9233.63078

**Published:** 2010

**Authors:** Adel H. Alsuhaibani

**Affiliations:** Department of Ophthalmology, King Abdulaziz University Hospital, King Saud University, Riyadh, Saudi Arabia

**Keywords:** Diagnosis, Facial Nerve Palsy, Keratopathy, Lagophthalmos, Management, Vision Loss

## Abstract

Development of facial nerve palsy (FNP) may lead to dramatic change in the patient's facial function, expression, and emotions. The ophthalmologist may play an important role in the initial evaluation, and the long-term management of patients with new-onset of FNP. In patients with expected temporary facial weakness, no efforts should be wasted to ensure proper corneal protection. Patients with permanent functional deficit may require combination of surgical procedures tailored to the patient's clinical findings that may require good eye comfort and cosmesis.

## INTRODUCTION

The facial nerve is vital for a person's well-being functionally, psychologically, and emotionally. Injury to the facial nerve (facial nerve palsy, FNP) leads to significant changes in the face, affecting many aspects of the patient's life. Ophthalmic manifestations present critical concerns for patients with lower motor neuron injury. The degree of FNP is variable. Patients with neurapraxia have temporary nerve conduction block owing to demyelination, which usually completely recover within 12 weeks. In axonotmesis, there is division of individual fibers leading to Wallerian degeneration distal to the injury. With intact endoneural tubes, axons reinnervate their original motor and sensory targets. Neurotmesis refers to division of fascicles and epineurium, and reinnervation of original motor and sensory targets may not occur.^1^

## ANATOMICAL CONSIDERATIONS

The facial nerve contains approximately 10,000 nerve fibers originating from four brainstem neuclie.^2^ These facial motor neuclie control muscles of facial experession, superior salivary nucleus innervating lacrimal and salivary glands, nucleus solitarius receiving fibers of taste from the anterior two-thirds of the tongue, and trigeminal sensory nucleus getting sensory fibers for a small portion of the external ear.^3^,^4^ The facial nerve leaves the brainstem at the lower border of the pons between the olive and inferior cerebellar peduncle and joined by the intermediate nerve. The facial nerve then enters to the internal acoustic meatus together with the eighth cranial nerve. The facial nerve travels through the fallopian canal within the petrous temporal bone for approximately 30-mm. The labyrinthine segment is the first and narrowest segment of fallopian canal where the facial nerve is susceptible to compression due to edema. During the canalicular course, the facial nerve has several branches. The first branch is the greater superficial petrosal nerve which exits at the geniculate ganglion and carries lacrimal and palatine secretory fibers to the pterygopalatine ganglion. Small branchs to the stapedius muscle and chorda tympani comprose the two branches.

Once it has exited the fallopian canal at the stylomastoid foramen, the facial nerve gives off several rami before it divides into its main branches. The facial nerve crosses lateral to the styloid process and penetrates the parotid gland. The nerve lies in a fibrous plane that separates the deep and superficial lobes of the parotid gland. In the parotid gland, the nerve divides into two major divisions, the superiorly directed temporal–facial and the inferiorly directed cervicofacial branches. After the main division, five major branches of the facial nerve exist: temporal (frontal), zygomatic, buccal, mandibular, and cervical.^5^,^6^ The facial nerve innervates 14 of the 17 paired muscle groups of the face on their deep side. The three muscles innervated from other sources are the buccinator, levator anguli oris, and mentalis muscles. The temporal branch of the facial nerve exits the parotid gland and runs within the superficial musculoaponeurotic system (SMAS) over the zygomatic arch into the temple region. The frontal branch enters the undersurface of the frontalis muscle and lies superficial to the deep temporalis fascia. To avoid injury to the frontal branch during elevation of facial flaps, the surgeon should elevate either in a subcutaneous plane or deep to the SMAS. The temporal branch supplies a part of the frontalis muscle and superior parts of the orbicularis. The zygomatic branch innervates frontalis and orbicularis oculi.

## ETIOLOGIES OF FACIAL PARALYSIS

Facial paralysis may occur due to various causes. Facial nerve injury resulting from a lesion above the geniculate ganglion presents with more severe ophthalmic symptoms because lacrimal secretion and orbicularis closure are involved.

### Idiopathic (Bell's Palsy)

The annual incidence of Bell's palsy in a population of 100,000 people is around 20 afflicted individuals.^7^,^8^ Bell's palsy most commonly occurs in the 15- to 45-year-age group and is much less common in children below 10 years.^9^ Bell's palsy is the most common cause of facial paralysis and is a diagnosis of exclusion. There may be some distinguishing characteristics in the history. These include an abrupt-onset with complete and unilateral facial paralysis within 24–72 h, as well as ipsilateral periauricular numbness or pain, decreased taste sensation, decreased production of tears, and/or hyperacusis. The usual course results in symptoms resolving spontaneously without the treatment within 6-month time. Some studies suggest the herpes virus as a cause, specifically herpes simplex virus type I (HSV 1), although no significant benefit has been shown from the use of acyclovir in the treatment of patients with Bell's palsy.^10^–^12^

### Trauma

The second most common cause of FNP is trauma,^13^ which may include blunt, penetrating, or iatrogenic trauma. Most commonly, traumatic injuries to the facial nerve are caused by temporal bone fractures. In temporal bone fractures, patient with immediate-onset of complete FNP may have poor recovery. On the other hand, patients with incomplete paralysis may have almost complete recovery.^14^ Blunt trauma usually leads to nerve edema and contusion and only rarely causes nerve transection. Gunshot wounds to the temporal bone may cause FNP 50% of the time with direct nerve transection or injury secondary to kinetic energy dissipated from the projectile bullet or bony fragments. Facial nerve injury during middle ear or mastoid surgery occurs with less than 1% in frequency and most commonly occurs in the tympanic segment of the nerve, as it is the most common site for fallopian canal dehiscence.^15^

### Infection

Geniculate ganglionitis (Ramsay-Hunt syndrome) caused by herpes zoster is classically associated with zoster vesicles on the ear, in the external auditory canal or tympanic membrane. Other associated Ramsay-Hunt syndrome symptoms include tinnitus, hearing loss, nausea, vomiting, vertigo, and nystagmus symptoms due to the proximity of the eight cranial nerve in this area. Compared with Bell's palsy patients, patients with Ramsay-Hunt syndrome often have more severe paralysis at onset and are less likely to recover completely.^16^ Lyme disease, tuberculous chronic HIV, polio, mumps, and leprosy are some of the infectious causes of FNP.^17^ Complications and sequelae of otitis media can lead to FNP. The risk exists for acute suppurative, effusive, malignant, and chronic otitis media. The mechanism is still unknown; however, it is assumed to be due to inflammatory edema and toxins and rarely due to actual compression of the nerve from, for example, cholesteatoma.^18^

### Neoplasms

Involvement of multiple cranial nerves, slow-onset of symptoms, no return of function after 6 months, and recurrent ipsilateral palsy are suggestive of a neoplastic cause of FNP. The most common tumor affecting facial nerve function is the acoustic neuroma. The most common tumor of the facial nerve itself is the facial schwannoma. In the presence of extratemporal tumoral process, FNP is rare. In such cases, workup for a possible parotid mass is indicated. Facial schwannomas can clinically resemble the more common acoustic neuromas. However, with facial schwannomas, a history of facial spasm often precedes the FNP. With improvement in the surgical removal of acoustic neuromas, 90% of the patients enjoy good facial nerve function (grades I–II) 1 year after surgery.^19^ Malignant parotid neoplasms are another fairly common cause of FNP.^20^ This may occur from direct nerve invasion or secondary to sacrifice of the facial nerve to achieve complete tumor extirpation. Nasopharyngeal carcinoma may affect the spheno-palatine ganglion or cavernous sinus and cause isolated tear deficiency associated with sixth nerve palsy.^17^

### Congenital

Facial palsy in a newborn may result from congenital to traumatic causes. Various syndromes may present with FNP that may include Moebius syndrome, Goldenhar syndrome, DiGeorge syndrome and CHARGE syndrome. Congenital palsies are most optimally treated later in childhood because improved cosmesis often requires muscle transfers and fascial slings. In contrast to older adults, children have better skin turgor and therefore rarely need protective ophthalmic care as eye closure is usually achievable despite impressive FNP. Birth trauma can result from compression molding while going through the birth canal or forceps use. Prognosis is often favorable, and nerve regeneration may spontaneously occur.^21^

### Miscellaneous

Systemic and metabolic disorders such as diabetes mellitus, hypertension, amyloidosis, and sarcoidosis may lead to FNP. Multiple sclerosis, Guillain-Barre syndrome, myasthenia gravis, and cerebrovasclar accident comprise some neurological causes of FNP.

## CLINICAL EVALUATION

**Figure 1:**
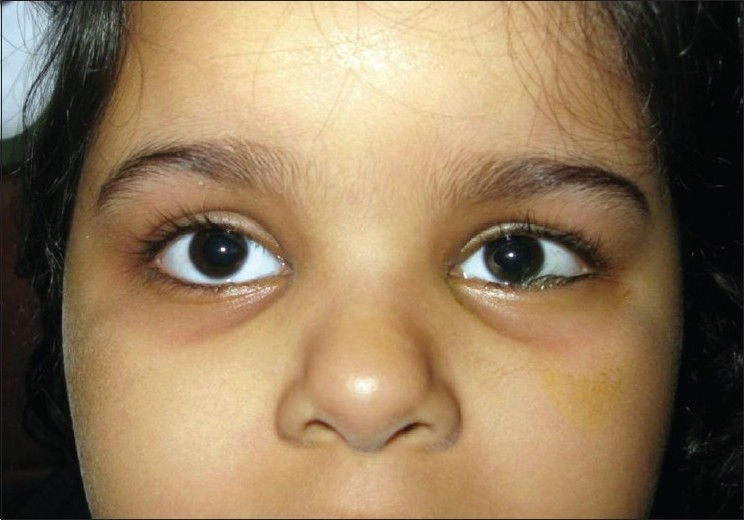
An 8-year-old girl with left-sided facial paralysis and left lower eyelid entropion. The patient developed left side facial paralysis as a complication of otitis media 6 months before presentation

Patients with FNP may typically present with brow ptosis, lagophthalmos, ectropion, and exposure keratopathy along with nasal alar collapse, nasolabial flattening, drooping of the corner of the mouth, and drooling. Tearing and synkinetic facial movements secondary to aberrant regeneration are other common complaints of patients with a history of FNP. Children with FNP may present with lower eyelid entropion^22^ [Figure 1].

An important part of the evaluation is the examination of corneal sensation along with other cranial nerves. Otoscopy needs to be performed to look for masses or vesicles in the auditory canal and conchal bowl. The severity of the clinical signs is variable. The House-Brackmann grading system^23^ has historically been used to categorize the severity of paralysis [Table 1].

**Table 1:** The House-Brackmann grading system

Grade I: Normal
Normal facial function in all areas.
Grade II: Mild dysfunction
*Gross*: Slight weakness noticeable on close inspection; may have slight synkinesis.
*At rest*: Normal symmetry and tone.
*Motion*: Forehead, moderate-to-good function.
*Eye*: Complete closure with minimal effort.
*Mouth*: Slight asymmetry.
Grade III: Moderate dysfunction
*Gross*: Obvious but not disfiguring difference between two sides with effort. Noticeable but not severe synkinesis, contracture or hemifacial spasm, or both.
*At rest*: Normal symmetry and tone.
*Motion*: Forehead, slight-to-moderate movement.
*Eye*: Complete closure with effort.
*Mouth*: Slightly weak with maximal effort.
Grade IV: Moderately severe dysfunction
*Gross*: Obvious weakness or disfiguring asymmetry, or both.
*At rest*: Normal symmetry and tone.
*Motion*: Forehead, none.
*Eye*: Incomplete closure.
*Mouth*: Asymmetric with maximal effort.
Grade V: Severe dysfunction
*Gross*: Barely perceptible motion.
*At rest*: Symmetry.
*Motion*: Forehead, none.
*Eye*: Incomplete closure.
*Mouth*: Slight movement.
Grade VI: Total paralysis
No movement

Laboratory studies such as rapid plasma reagin (RPR) and/or venereal disease research laboratory (VDRL) test or fluorescent treponemal antibody absorption (FTA-ABS) test, and HIV screening may be requested depending on the clinical scenario. Imaging studies are required in patients with atypical presentation or when the FNP appears central to rule out a tumor or vascular compression.

## MANAGEMENT OF OPHTHALMIC MANIFESTATIONS AND CONSEQUENCES OF FACIAL PALSY

Patients with FNP may require a multidisciplinary approach for proper management. In the majority of patients with FNP, there may be spontaneous recovery of good nerve function within a few months of its onset. During the period of recovery, temporary measures may be utilized to ensure good eye care. In some patients, the recovery of the nerve function is not expected such as removal of the facial nerve during removal of malignant parotid neoplasms removal. In these situation, starting with the permanent measures is recommended.

### Temporary measures

The aim of the temporary measures is to keep the involved eye well-lubricated awaiting for recovery of the nerve function. In most cases, topical ocular lubrication (with artificial tears during the day and lubricating ophthalmic ointment at night, or occasionally ointment day and night) is sufficient to prevent exposure keratopathy. Occluding the eyelids with tape or by applying a patch for 1 or 2 days may help heal corneal erosions. Care must be taken to prevent worsening of the abrasion with the tape or a patch by ensuring that the eyelid is securely closed. External eyelid weights are available to improve mechanical blink.^24^,^25^ The weights are attached to the upper eyelid with an adhesive and are available in various colors. Injection of hyaluronic acid gel in the prelevator aponeurosis region and/or pretarsal region may be useful in patients who are poor surgical candidates and/or as a temporizing measure.^26^

Temporary tarsorrhaphy with suture or cyanoacrylate glue is another practical option for providing eye protection and can be opened at a later date.^27^ Temporary tarsorrhaphy may be performed laterally, centrally, or paracentrally (lateral to the lacrimal punctum). Paracentral tarsorrhaphy provides good closure without affecting the temporal visual field. Tarsorrhaphy can also be used as part of permanent measures. Lower-lid ectropion or droop can temporarily be helped by applying tape below the lid margin in the center of the lower lid to pull the lid laterally and upward to anchor on the orbital rim. In some cases, botulinum toxin can be injected transcutaneously or subconjunctivally at the upper border of the tarsus to produce temporary ptosis for corneal protection.^28^

**Figure 2:**
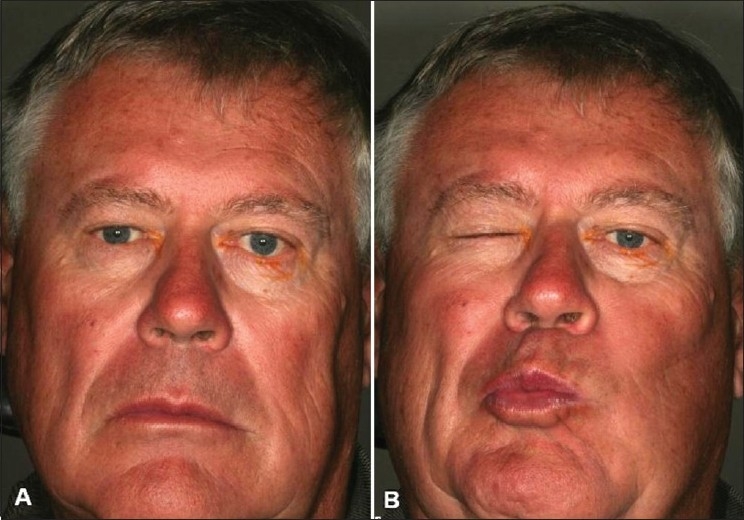
The right eye is smaller than the left eye (A). The right eye became even smaller due to aberrant regeneration resulting in co-contraction of orbicularis oculi with movement of the mouth (B)

Synkinetic eyelid movement secondary to aberrant regeneration is one of the long term complications of FNP [Figure 2]. The actual incidence is unknown, but is more common in patients who suffer the severe form of nerve injuries, with complete or near complete facial paralysis. It appears most commonly after 24–39 weeks following onset of FNP.^29^ Typical movements include blinking with oral movement, intermittent ptosis (eyelids close when the mouth is closed from misdirection of fibers of the orbicularis oris into the orbicularis oculi), and facial spasm with eyelid closure.^30^,^31^ Botulinum toxin may help in relaxing the synkinetic movements secondary to aberrant regeneration.^32^,^33^ Only very low doses are required due to the denervation hypersensitivity,^34^ though the results are not as satisfying as in patients with Bell's palsy and in patients with idiopathic hemifacial spasm.

### Surgical options for permanent measures

#### Correction of lagophthalmos

**Figure 3:**
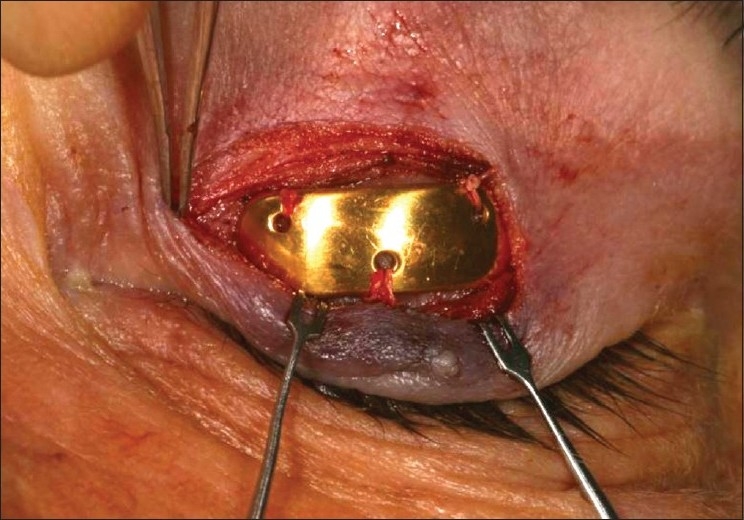
Left upper eyelid pretarsal gold-weight implantation for management of lagophthalmos

Implantable devices have been used to restore dynamic lid closure in cases of severe, symptomatic lagophthalmos. These procedures are best for patients with poor Bell's phenomenon and decreased corneal sensation. Gold or platinum weights,^35^,^36^ palpebral springs,^37^ or facial slings^38^ can be inserted into the eyelids. Pretarsal gold-weight implantation is most commonly performed^39^ [Figure 3]. The weight allows the upper eyelid to close with gravity. Therefore, patients must sleep with their head slightly elevated. These implants are inert and composed of 99.99% pure gold or platinum. Sizes range from 0.6 to 1.8 g. They are easily removed if nerve function returns. Complications include migration of the implant, inflammation, allergic reaction, extrusion, or induced astigmatism.^40^–^42^

Lid retraction is a common sequela of facial paralysis. Lid retraction is presumed to be secondary to unopposed action of levator superioris. However, Aramideh *et al*.^43^ suggested thixotrophy as a cause of lagophthalmos. They described thixotrophy as stiffness of a striated muscle and formation of tight crossbridges between the actin and myosin filaments within muscle fibers causing stiffness of the muscle. Eyelid retraction must be addressed before the placement of upper eyelid weight. Numerous procedures are available for lowering the upper eyelid, and the choice may depend on the degree of retraction. For mild retraction, Muller's muscle excision is sufficient. Levator recession with or without a spacer material may be required.^44^

Transposition of the temporalis muscle can be used to reanimate the face and provide lid closure using the action of fifth cranial nerve.^45^ Strips from the muscle and fascia are placed in the upper and lower lids as an encircling sling. Patients initiate movement by chewing or clenching their teeth. Reinnervation of the facial nerve by means of facial nerve grafting or hypoglossal–facial nerve anastomosis can be used in cases of clinically significant permanent paralysis to help restore relatively normal function to the orbicularis oculi muscle or eyelids.^46^,^47^

#### Correction of lower eyelid droop and ectropion

A lateral tarsal strip procedure can be performed to correct horizontal lower-eyelid laxity and to improve apposition of the lid to the globe.^48^ Mid-face lift with or without spacer insertion in the lower lid may be carried out in patients with severe lower eyelid malpositions. This procedure may help address the facial asymmetry with vertical face lifting while avoiding an unnatural tension on the skin.^49^

#### Brow ptosis and blepharoplasty

Brow ptosis may be corrected with a direct brow lift. Internal brow lift with anchoring sutures to the brow periosteum through the upper eyelid blepharoplasty or endoscopic brow lifts are some other surgical options.^50^ After the correction of the brow ptosis, excessive upper eyelid skin may be excised with a conservative blepharoplasty to avoid aggravating corneal exposure and lagophthalmos.

### Epiphora

#### Reflex tearing from dry eye

Reflex epiphora can be improved by keeping the eye well-lubricated with eye lubricants, reduction of the palpebral aperture and improvement of eyelid closure.

#### Paralytic ectropion

Lacrimal pump function failure occurs due to orbicularis paralysis and loss of normal blinking. Eyelid laxity associated with FNP exacerbates the epiphora. In these cases, lateral eyelid tarsal strip procedure may improve tear drainage. Dacryocystorhinostomy and Jones tube insertion may be required to decrease the resistance to tear outflow.

#### Hypersecretion/aberrant innervation of lacrimal gland

Central lesions causing FNP may lead to “crocodile tear” when regenerating fibers of the chorda tympani grow down the lacrimal secretory neural pathway (misrouting of postganglionic parasympathetic secretomotor fibers). This hypersecretion can be successfully treated with botulinum toxin A injections into the lacrimal gland.^51^ The effect is temporary and requires repeat treatments. Overtreatment may result in ptosis, strabismus, dry eye, or excessive lagophthalmos.^52^ Transconjunctival intraglandular injection provides a direct and effective approach in the treatment of crocodile tears.^53^

## CONCLUSION

Facial paralysis may be a difficult disease process to diagnose and manage. It is crucial to recognize and treat the potentially life-threatening underlying causes. Patients with FNP may require multidisciplinary approach for the proper management. Ophthalmologists may provide a significant contribution by providing eye protection and esthetic improvement for the periorbital area.
